# Toward a Dynamic Probabilistic Model for Vestibular Cognition

**DOI:** 10.3389/fpsyg.2017.00138

**Published:** 2017-02-01

**Authors:** Andrew W. Ellis, Fred W. Mast

**Affiliations:** Department of Psychology, University of BernBern, Switzerland

**Keywords:** mental imagery, mental simulation, spatial perspective taking, spatial cognition, self-motion perception, particle filters, computational modeling

## Abstract

We suggest that research in vestibular cognition will benefit from the theoretical framework of probabilistic models. This will aid in developing an understanding of how interactions between high-level cognition and low-level sensory processing might occur. Many such interactions have been shown experimentally; however, to date, no attempt has been made to systematically explore vestibular cognition by using computational modeling. It is widely assumed that mental imagery and perception share at least in part neural circuitry, and it has been proposed that mental simulation is closely connected to the brain’s ability to make predictions. We claim that this connection has been disregarded in the vestibular domain, and we suggest ways in which future research may take this into consideration.

## Introduction

In contrast to other sensory modalities, the vestibular sense has been widely neglected by cognitive scientists. Vestibular information was until recently considered in the context of posture and balance, and was therefore associated with reflexive behavior. However, Angelaki et al. (2009) highlight the fact that the focus of research has shifted to include the involvement of the vestibular system in spatial cognition. It is now known that several cortical areas are under vestibular control (see Lopez et al., 2012 for a meta-analysis of vestibular neuroimaging studies) and recent behavioral studies provide evidence of bi-directional interactions between higher-level cognition and vestibular processing (see Mast et al., 2014 for a review).

Two recent experiments have provided interesting contributions to vestibular cognition. Deroualle et al. (2015) showed that vestibular sensory input has an effect on spatial perspective taking; if the participants were physically rotated in a direction congruent with the direction of a mental self-rotation, they were faster in giving correct responses. This suggests that sensory information about self-motion is involved in cognitive operations required when taking another person’s perspective. Interestingly, Nigmatullina et al. (2015) demonstrated an effect in the reverse direction; imagined self-motion affects the sensory processing of physical rotations. Specifically, they showed that imagined self-motion influences both the onset of the vestibulo-ocular reflex (VOR), and the perception of self-motion. These two studies compellingly demonstrate that vestibular processing is nested and intertwined with cognitive processes. To date, however, empirical findings in vestibular cognition remain a set of rather loosely connected phenomena (Mast et al., 2014), and it is unclear how interactions between mental simulations and lower-level processing of vestibular information can be embedded within a coherent theoretical framework. This is rather surprising, since there is a long tradition of computational modeling in vestibular research. Indeed, due to its relatively well-understood pathways at the subcortical level, the system is particularly amenable to mathematical modeling, and a wealth of knowledge has been ascertained in basic mechanisms of vestibular processing (Merfeld et al., 1999; Zupan et al., 2002; Angelaki et al., 2004; Angelaki and Yakusheva, 2009; Laurens et al., 2013) (see MacNeilage et al., 2008; Selva and Oman, 2012 for overviews).

We claim that dynamic probabilistic models offer a computational and theoretical framework for vestibular cognition. There has been an increasing focus on Bayesian inference in computational approaches to cognitive modeling (Griffiths et al., 2008, 2012; Chater and Oaksford, 2013); higher order cognitive processing is thought to require structured representations, which can be implemented as Bayesian Networks (Griffiths et al., 2010; Kwisthout et al., 2016). The Bayesian approach has yielded successful attempts at explaining a variety of phenomena, from categorization of objects (Kemp et al., 2007) and counterfactual reasoning (Lucas and Kemp, 2012) to perceptual switching when viewing bi-stable stimuli (Gershman et al., 2012). Similar computational models have been proposed for lower-level processing in several sensory modalities (Fetsch et al., 2012). In fact, probabilistic models are well established in the vestibular domain, given the noisy and ambiguous nature of the sensory afferent signals and the fact that vestibular afferents are combined with proprioceptive signals at a very early level in the brain (Angelaki et al., 2009). Bayesian models can provide the optimal solution to the problem of combining information from multiple sources. We have previously argued that vestibular cognition can be viewed as being similar to vestibular sensory processing, albeit in an oﬄine mode of processing (Mast and Ellis, 2015). In this paper, we claim that in order to investigate how higher-level cognition and lower-level vestibular processing interact, it is useful to consider cognitive influences as a hierarchical extension within a Bayesian framework. Given that the vestibular system processes sensory signals related to motion, further insight can be gained by considering dynamical models.

## Dynamic Probabilistic Models

Bayesian inference gives a prominent role to prior information or knowledge. On the one hand, prior knowledge may be built into the system by evolution, or acquired during ontogeny. These types of priors may reflect stable statistical properties of the environment, and may be relatively inflexible. One particularly well-known example is the prior belief that the head is usually aligned with the gravitational vertical (Eggert, 1998), which has been shown to partly explain the misperception of the visual vertical (De Vrijer et al., 2009). On the other hand, priors may reflect properties of a rapidly changing environment, and may therefore be flexible. It is precisely this kind of flexible prior belief that is interesting for the study of vestibular cognition.

**FIGURE 1:**
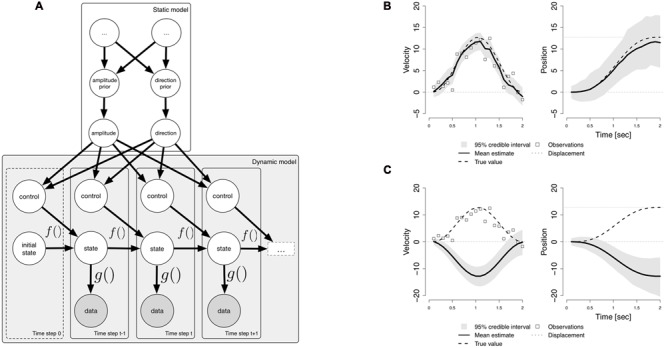
**(A)** A hierarchical dynamic probabilistic model for sensory inference. The lower part (Dynamic model) shows a representation of a basic dynamic probabilistic model used for sensory inference. The circles represent random variables. Shaded circles are observed (sensory data); unshaded circles represent latent variables. Bayesian inference is performed at each time step in order to estimate the state by combining the prior, given by the function f of the previous state and a control input, with the data, which depends on the state through a function g. The upper part (Static model) of the figure shows a hierarchical extension to the dynamic model. These variables do not change at the time scale of the state variables, and are used in order to construct a dynamic prior for inference. The variables at the highest level are unnamed (…), indicating that the model can be extended. **(B)** The results of inferring the velocity and position. **(C)** The same generative model being used to perform a simulation.

In order to process dynamic vestibular sensory information, the brain must possess a specific type of generative model; this can be described as a state-space model (Karmali and Merfeld, 2012), which allows the brain to infer the values of unobservable state variables, such as the position and velocity of the head, based on a sequence of noisy sensory input data from the semi-circular canals (SCC). In addition, the brain is able to predict the sensory consequences of active head movements (Cullen, 2012), and this may be implemented as an active control input in the generative model. A state space model can also be described as a dynamic probabilistic model (Bishop, 2006). The lower part of **Figure 1A** (Dynamic model) shows a graphical representation of such a model. The variables are represented by nodes (circles), with the arrows indicating stochastic relationships between variables. The generative model consists of a process model *f*, which describes the evolution of the latent state variables, and an observation model *g*, which describes the dependence of the SCC afferent signals (data) on the state variables. The process can implement Newton’s laws of motion. The data nodes are shaded, indicating that they are observed during sensory inference, whilst the state variables are open, indicating that they must be inferred. At each time step, the state variable depends on the state at the previous time step, and a control input, which consists of a known acceleration. This control input enables the brain to predict its head velocity and position. Knowledge about the body’s kinematics is useful in order to compute the expected sensory consequences (re-afference) of active movements (Vonholst and Mittelstaedt, 1950).

The exact nature of the dependence of the control input on its parent nodes is encoded in the edges leading from the higher-level nodes (Static model) in **Figure 1A**. These variables represent parameters of a motion episode. In the case of a sinusoidal rotation of the head at a given frequency, the brain may compute the known acceleration based on the amplitude and direction of motion. These variables, if they must also be inferred, can be given prior distributions. The direction of motion can be modeled as a binary random variable (either to the left or to the right), and the prior on motion direction can be given a uniform distribution, indicating that both directions are equally likely, or could indicate a preference for either direction. The higher level variables in the static model thus represent parameters that do not change during the course of a motion event. Active head movements are performed in order to achieve a goal at a more abstract functional level, e.g., turning one’s head in order look at something, or in order to achieve a communicative goal (Carriot et al., 2014). The hierarchical model can be extended in order to incorporate such higher-level intentions and goals (see the unnamed uppermost nodes).

The exogenous control input is usually interpreted as being derived from a motor action, in the form of an efference copy. However, this input may represent any available knowledge about statistical properties of the environment, or it can be derived from covert actions (Pezzulo and Castelfranchi, 2009). In the context of self-motion, this means that the brain is able to construct a model of the kinematics, and of the expected vestibular sensory afferents. The generative model opens the gates for higher-level cognition, and in particular spatial perspective transformations and imagined self-motion.

In order to gain an understanding of the involvement of cognition in lower-level sensory processing, it is interesting to consider how inference is performed in a dynamic probabilistic model, and the role of predictions. A frequently used inference algorithm is particle filtering (Doucet et al., 2000, see Speekenbrink, 2016 for a recent introduction). Particle filtering starts from an initial state, and then recursively applies a sequence of computations. At each time step, a prediction is first made for the velocity and position, based on the previous state and known input. After predicting the state, the SCC data are incorporated by computing how plausible the sensory signal is, given the belief about the state. When combined, these two steps implement sequential Bayesian inference; a prior belief is computed by prediction, and then the likelihood is combined with the prior to obtain a posterior estimate. By choosing a specific kinematic model, one can specify a strong prior belief that one is either at rest or in motion. The ability to flexibly choose a kinematic model is essential in order to predict the expected sensory consequences of active motion (Cullen et al., 2011; Cullen, 2012). Sensory processes play a major role in predicting the future, and sensory measurements serve to correct the predictions. **Figure 1B** shows the results of inferring the velocity and position, based on noisy sensory input from the SCC during a 2 s leftward sinusoidal rotation of the head, using a kinematic model which incorporates prior knowledge about head acceleration. Using this knowledge, the model can accurately estimate the true velocity (left), and the true position (right).

## From Vestibular Processing to Higher Cognition

Given this rather constructive nature of vestibular sensory processing, and the fact that this can explain velocity storage (Karmali and Merfeld, 2012) and the illusion of translation during off-vertical axis rotation (Laurens and Droulez, 2007), we suggest that the act of imagining self-motion is related to the generative activity of the vestibular network. A number of recent articles have alluded to a link between mental imagery and the brain’s predictive mechanisms (Grush, 2004; Moulton and Kosslyn, 2009; Clark, 2012; Gambi and Pickering, 2015), but few studies have addressed this issue within a computational framework, with a view to providing a mechanistic explanation of how mental imagery should be related to predictions. A notable exception is Penny et al. (2013), who demonstrated that various computations necessary for spatial cognition can be performed within the same dynamical probabilistic model. To our knowledge, there have been no previous attempts to link imagined self-motion to vestibular processing in a computational framework.

In order to link higher cognition to the model for sensory inference, we can consider the extension to the basic dynamic model presented in **Figure 1A**. What is required for higher-level cognition is the ability to incorporate variables representing higher level priors into the basic dynamical model (Griffiths et al., 2008). On the one hand, the model can infer the values of these higher level direction and amplitude nodes (Andrieu et al., 2010). On the other hand, the generative model may be used to perform the different types of computations described in Penny et al. (2013), in the form of Monte Carlo simulations, i.e., by running the dynamic probabilistic model without incorporating sensory data. This amounts to sampling from the prior distributions (Berkes et al., 2011), albeit a dynamically constructed prior, given by the process model. Static variables must be either fixed at certain values, or sampled from their prior distributions, and, crucially, incoming sensory data must be ignored. **Figure 1C** shows the result of running a Monte Carlo simulation using the same dynamic probabilistic model as in **Figure 1B**. However, in this case, the sensory data, which indicate leftward motion, are not incorporated, and the direction is to the right. This allows an implementation of a ‘mental simulation’ (Pezzulo et al., 2011).

## Interactions Between Imagined and Perceived Self-Motion

**FIGURE 2:**
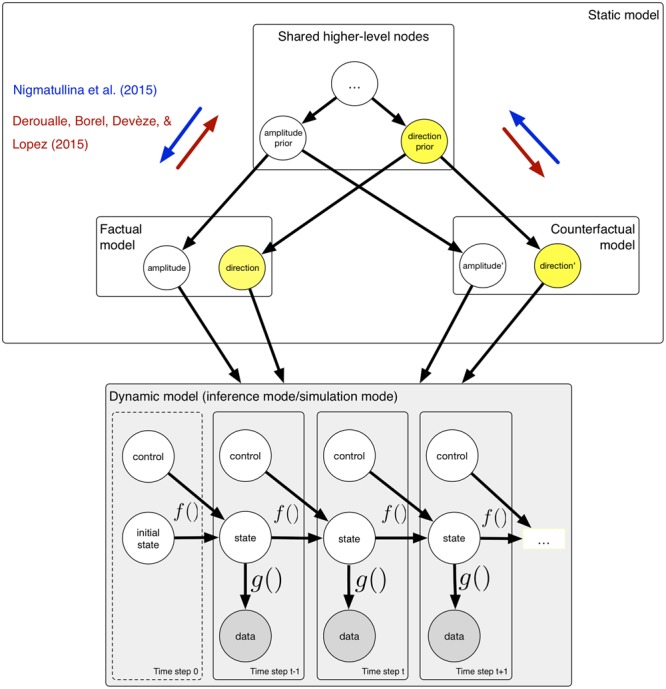
**A computational framework that explains how interaction between mental simulation and perception occurs.** For the purpose of mental simulation, a twinned counterfactual model is constructed, which shares both higher-level and lower-level nodes with the factual model used for sensory inference. The dynamic model may be run in either inference mode, or in simulation mode. During inference mode, sensory data are incorporated; in simulation mode, sensory data must be ignored. The factual and counterfactual models reflect the fact that for simulation, the variables do not represent the state of the world. Interactions between sensory and simulation models occur via the shared higher-level nodes.

The studies by Deroualle et al. (2015) and Nigmatullina et al. (2015) suggest that simulated self-motion and self-motion perception share common processes. We claim that the computational framework described above can be used to investigate these interactions in terms of probabilistic computations. This need not entail that the brain uses the same generative model for simulation and sensory inference; indeed, in the experiment of Deroualle et al. (2015), participants were required to perform a simulated rotation whilst simultaneously processing sensory data, which implies that the brain must either use spatially separated models, or implement a time-sharing mechanism via oscillations (Lisman and Buzsáki, 2008) in order to separate the two processing streams. It is worthwhile considering that mental simulations are performed under the counterfactual assumptions that the contents of one’s mental activity do not reflect the current state of the world. For the purpose of modeling interactions, it is sufficient to assume that the higher-level nodes used to construct the kinematic model required for mental simulations are shared, without committing to any specific lower-level implementation. **Figure 2** illustrates the idea that the low-level dynamic graphical model can be used in sensory inference mode or in simulation mode. We propose that the brain may construct a ‘twinned’ counterfactual model (Koller and Friedman, 2009) for the purpose of a mental simulation; this model shares both higher-level components with the factual model used for sensory inference, and re-uses lower-level variables in order to perform realistic simulations.

In Nigmatullina et al.’s (2015) experiment, participants were required to imagine themselves rotating, prior to making a judgment about their actual motion. This might have resulted in the direction variable either being clamped or having a strong prior, with the effect that, during subsequent sensory inference, the participants may have inadvertently used this strong prior. Participants were faster to detect motion that was congruent with the direction they had previously imagined, and slower to detect motion in the incongruent direction, and the same result was obtained for the VOR onset. Intriguingly, this pattern of response times is surprisingly similar to that what one might expect to find if participants were using prior knowledge to anticipate a given direction (Leite and Ratcliff, 2011; Mulder et al., 2012). It is likely that mental imagery exerts an influence on sensory inference by ‘biasing’ higher-level variables, in a similar manner to anticipation of sensory events. The direction of this influence is shown in **Figure 2** by the blue arrows. Imagining motion led the participants to unintentionally create an expectation for subsequent actual motion. This makes the claim that mental imagery is related to prediction explicit, and explains this connection in the context of a coherent computational framework. A similar explanation may be proposed for the experimental findings in Deroualle et al. (2015); here, the direction of interaction is reversed, indicated by the red arrows in **Figure 2**. Taken together, when required to simultaneously perform simulation and sensory inference using shared higher-level nodes in a probabilistic model, there is cross-talk.

## Neuronal Implementation

Brooks and Cullen (2013) report that the cerebellum implements a forward model, and at the level of the vestibular nuclei, the expected afferent signals (re-afference) are suppressed. Furthermore, thalamic vestibular neurons also distinguish between active and passive head movements (Lopez and Blanke, 2011). It is not clear whether higher cognitive processes, such as imagery or spatial perspective taking, involve such low levels; however, there exist both direct and indirect connections between the vestibular nuclei and parieto-insular cortical (PIVC) areas known to process vestibular signals (Kirsch et al., 2016). Furthermore, human subjects are able to suppress the VOR by imagining a head-fixed target (Jones et al., 1984). Thus, seemingly low-level reflexes require considerable flexibility, depending on the organism’s goals and intentions.

A broad overview of the cortical representation of vestibular information is given in Lopez and Blanke (2011) and Lopez et al. (2012). Vestibular neuroimaging studies involving cognitive aspects are still scarce. In an fMRI study on imagined self-rotation, zu Eulenburg et al. (2012) found activation in regions involved in spatial processing, but failed to find activation of PIVC. It is important to note, however, that conditions using GVS and caloric vestibular stimulation (CVS), which are usually used in lieu of actual physical rotation, entail several problems (Klingner et al., 2016). The authors point out that, due to the inappropriateness of GVS and CVS, most imaging results of the vestibular system may actually represent strong multisensory prediction errors.

## Implications for Future Research

The close connection between mental simulation and sensory inference, through the use of common components of a generative model, has strong implications for future experiments in vestibular cognition. Special care must be taken to compare experimental conditions in which participants perform cognitive tasks, such as mental imagery or spatial perspective taking, with control conditions in which participants use prior information in order to form strong expectations about sensory inputs. Furthermore, neuroimaging studies are required that specifically investigate expectations of head and whole body movements, the distinction between active and passive movement, egocentric spatial transformations and the accumulation of vestibular sensory evidence for decision-making. This will allow researchers to disentangle the higher-level cognitive operations from predictive processing during on-line sensory inference.

## Conclusion

We propose that dynamical probabilistic models will help to advance the field of vestibular cognition. These types of models have been successfully used in computational approaches to lower-level vestibular processing, and represent the state of the art in robotics and machine learning. Recently, they have been applied to higher-level cognition. We claim that the vestibular system is ideally suited for investigating interactions between higher-level cognition and lower-level sensory processing, and that these interactions can be understood in terms of probabilistic computations performed by the brain in order to run realistic counterfactual simulations of self-motion. Probabilistic computational modeling in combination with thorough experimentation will bring vestibular science to the next stage and bridge the gap to cognitive operations and foster new clinical approaches.

## Author Contributions

Both authors have made a substantial, direct and intellectual contribution to the work, and approved it for publication.

## Conflict of Interest Statement

The authors declare that the research was conducted in the absence of any commercial or financial relationships that could be construed as a potential conflict of interest.
